# Factor Structure of the Short-Form of Center for Epidemiological Studies Depression Scale for People With Physical Disabilities

**DOI:** 10.3389/fpsyt.2021.536499

**Published:** 2021-04-14

**Authors:** Eun-Young Park

**Affiliations:** Department of Secondary Special Education, College of Education, Jeonju University, Jeonju, South Korea

**Keywords:** factor structure, Center for Epidemiological Studies Depression scale, people with physical disabilities, validity, reliability

## Abstract

This study aimed to examine the factor structure of the short-form of Center for Epidemiological Studies Depression Scale (CES-D-11) using confirmatory factor analysis (CFA). We extracted data from 670 people with physical disabilities (PWPD) from the Korea Welfare Panel. To investigate the model fit regarding factor structure, a one-factor model, four-factor model, and four-factor within bifactor model, as reported in previous studies, were examined using CFA, and goodness-of-fit indices were compared. As a result of the analysis, the four-factor model and the four-factor within bifactor model satisfied the criteria of correspondence with goodness-of-fit indices. Reliability of the four individual factors ranged from 0.722 to 0.834, indicating acceptable reliability. Validity and reliability of the four-factor within bifactor structure was confirmed through CFA and reliability analysis. In future studies using the CES-D-11 to measure depression in PWPD, comparison between four sub-factors and total scores might be possible.

## Introduction

Research has indicated that people with physical disabilities or mental dysfunction experience depression more often than the general population (1). Due to the loss of physical functioning, people with physical disabilities (PWPD) can experience multi-faceted environmental effects like a failure to adequately cope with such changes, lower social participation, low self-esteem due to disability, and a lack of social support (2, 3). This is consistent with the view that physical disability represents a source of enduring social stress (4). Furthermore, chronic stress and loneliness are well-known risk factors for high levels of distress and depression (5).

Since PWPD have such a high risk of depression, their mental health issues must be considered in more depth. To better understand disability and depression, the ability to accurately measure depression for PWPD must take precedence. The Center for Epidemiologic Studies Depression Scale (CES-D), developed by Radloff (6), is a 20-item self-report scale that measures depression levels in the general population. It is one of the most widely used research tools worldwide (7, 8) and the reliability and validity of the CES-D has been established in several studies (9, 10).

As the measurement of depression is related to sociodemographic factors (11), studies have assessed the validity of the CES-D across different populations (8, 12, 13). The factor structure of the 20-item CES-D for Korean population was confirmed in various studies (14, 15). However, there is a lack of research investigating the psychometric properties of the CES-D for PWPD, a group with characteristics different from the general population and a high prevalence of depression. Despite the usefulness of the 20-item CES-D, it does not apply to respondents such as physically ill patients, the elderly, people with difficulty in reading or understanding the questions, and people having difficulty in answering a questionnaire with a larger number of questions. Recognizing that this could be a significant burden on respondents (16), interest in shortened versions of the CES-D has increased (17–19). Previous studies confirming the usefulness of the CES-D for PWPD used a 20-item scale; the validity of the CES-D-11, however, has not been confirmed in this population. The correlation between the CES-D-11 and CES-D-20 was 0.95, and four subfactor structures were identified through factor analysis (20). The validity of CES-D-11 in Korean population was reported (21). A Korean study confirming the invariance of measurement between gender and age groups of CES-D-11 showed that it fit well with the 4-factor model (22). Hoe et al. (22) confirmed the following four factors: depressed, positive affect, somatic, and interpersonal relations.

The classic method for validating a scale is factor analysis; therefore, this study's purpose was to examine the validity of the CES-D-11 factor structure for PWPD. The specific research questions addressed were: (1) what is the factor structure of the CES-D-11 for PWPD? and (2) what is the reliability of the CES-D-11 for PWPD?

## Methods

### Participants

This study used data from the Korea Welfare Panel to verify the factor structure of the CES-D-11 for PWPD. Ethical approval was not applicable to this analysis. The following were inclusion criteria: first, participants were registered as PWPD under the Health and Welfare Act. The registration of individuals with disabilities requires the diagnosis of disability by a doctor who can register the type of disability according to their diagnosis. Therefore, participants in this study were limited to those who were diagnosed with a physical disability by a doctor. Second, participants who consented were included in the data investigation process. There were no specific exclusion criteria, however, if the participants included in the sample did not consent to respond to the scale, they were excluded and replaced by other participants by following the sampling procedure. Of the 670 respondents, 307 (45.8%) were male and 363 (54.2%) were female. Regarding age distribution, most respondents were above 60 (79.6%). The highest percentage of participants had an elementary school education level (*n* = 280, 41.8%). There were significant differences in depression levels across gender, age, and education level for PWPD. Respondents who were female, above 60 years old, illiterate, and had an elementary school education showed higher depression levels (Table 1). Configural invariance was used to calculate the fit index according to gender, age, and education level. Due to the small number of participants, configural invariance for ages below 41 could not be completed. As shown in Table 1, fit indices in each group showed an acceptable level, except for the RMSEA value in the 41–60 years group, illiteracy group, and graduate middle school group.

**Table 1:** Score differences according to participants' general characteristics.

**Category**	***N* (%)**	**M**	**SD**	***t* or F**	***P***	**Fit index of four-factor model within group**		
						****χ^2^****	**CFI**	**RMSEA**
**Gender**								
Male	307 (45.8)	8.51	9.32	−3.528	<0.001	116.966^**^	0.947	0.082
Female	363 (54.2)	11.21	10.34			93.245^**^	0.970	0.063
**Age**								
Below 41	14 (2.1)	6.10^ab^	5.27	3.519	0.030	-	-	-
41~60	123 (18.4)	8.28^a^	10.59			84.563^**^	0.947	0.100
Above 60	533 (79.6)	10.47^b^	9.86			139.461^**^	0.959	0.071
**Education level**								
Illiteracy	116 (17.3)	12.90^a^	11.37	6.865	<0.001	86.518^**^	0.930	0.105
Elementary school	280 (41.8)	10.32^a^	9.48			104.921^**^	0.947	0.079
Middle School	102 (15.2)	9.70^ab^	9.91			79.024^**^	0.925	0.103
Above high school	172 (25.7)	7.61^b^	9.25			63.509^**^	0.970	0.063

** *p < 0.001; CFI, comparative fit index; RMSEA, root mean square error of approximation; Mean (^ab^) = not significantly different at p < 0.05 level*.

### Measure

Depression was measured using the CES-D-11. Scores ≥ 16 are considered an indicator of clinically significant depressive symptoms (23). Items were scored on a 4-point Likert scale ranging from 0 (*nearly or never*) to 3 (*most or all of the time*), with higher scores being considered as indicators of clinical depression. Items included poor appetite, felt as good as others, felt depressed, felt everything was an effort, troubled sleep, felt lonely, unfriendly people, enjoyed life, felt sad, others dislike me, and could not get going.

### Data Analysis

Confirmatory factor analysis (CFA) with maximum-likelihood estimation was conducted to determine the suitability of the CES-D-11 factor structure (24). The CES-D was originally developed using a four-factor structure; however, validity verification processes in subsequent studies suggested one-factor (25) and four-factor structures (26). Therefore, this study aimed to confirm the factor structure of CES-D-11 for PWPD by comparing one-factor and four-factor structures, as well as a bifactor structure, in order to determine the possibility of comparing total scores presented in previous studies. The unknown estimate uses a maximum likelihood method that assumes a multivariate normal distribution of measured variables (24). The goodness-of-fit index is usually used in several indices (27). In this study, χ^2^, relative sum index, and root mean square error were used as goodness-of-fit indices for the models. The χ^2^ test does not trust χ^2^ because researchers estimate the model from large data samples due to significant increases in the power of the sample; as the power increases, it is easy to reject models that explain its data well (28). CFI is a suggested value which improves the problem of the standard fit index and is considered a good model when the value is over 0.90 (29). Root Mean Square Error of Approximation (RMSEA) was used to examine residuals in the model and smaller values indicated better fit. Values below 0.08 are recommended as an acceptable level (30). Akaike information criterion (AIC) is useful for comparing two or more models. After factor analysis, the goodness of fit level of the CES-D-11 was examined. Data processing was performed using AMOS (ver. 24.0). After confirming factor structure, internal consistency of the CES-D-11 was examined using SPSS (ver. 24.0).

## Results

### CFA of the CES-D-11

Table 2 presents fit indices for each CES-D-11 model and measurement invariance according to gender. The first model posited that all items load on a single factor. In the second model, the items were hypothesized to load on four factors: depressed, positive affect, somatic, and interpersonal relations. Next, the bifactor model was examined. The fit indices supported the four-factor model for the CES-D-11 among PWPD. Although the four-factor model was identified as superior by the AIC value, the bifactor model also had strong goodness-of-fit indices. The results of the fitness test confirmed metric invariance, scalar invariance, uniqueness invariance, and factor variance/covariance invariance of the CES-D according to gender. Table 3 presents regression weights for the CES-D-11 four-factor model and four-factor within bifactor model. All paths showed significant coefficients at the 0.05 level.

**Table 2:** Comparison of three competing models of the CES-D-11 and measurement invariance.

**Category**		***df***	**χ^**2**^**	**CFI**	**RMSEA (LO 90**~**HI 90)**	**AIC**
Model	One-factor	44	471.406^**^	0.870	0.120 (0.111 ~ 0.130)	537.406
	Four-factor	38	139.546^**^	0.969	0.063 (0.052 ~ 0.075)	217.546
	Bifactor	40	155.285^**^	0.965	0.066 (0.055 ~ 0.077)	299.285
Measurement Invariance according to gender	Unconstrained	76	210.222^**^	0.960	0.051 (0.043 ~ 0.060)	-
	Metric invariance	83	221.323^**^	0.959	0.050 (0.042 ~ 0.058)	-
	Scalar invariance	104	300.846^**^	0.941	0.053 (0.046 ~ 0.060)	-
	Uniqueness invariance	79	211.122^**^	0.961	0.050 (0.042 ~ 0.058)	-
	Factor variance-covariance invariance	115	382.576^**^	0.920	0.059 (0.053 ~ 0.066)	-

** *p < 0.001; CFI, comparative fit index; RMSEA, root mean square error of approximation; AIC, akaike information criterion*.

**Table 3:** Regression weights of four-factor model.

**Category**	**Four-Factor Model**				**Category**	**Four-Factor model within Bi-factor**			
	**Items**	**B**	**SE**	**CR**		**Items**	**B**	**SE**	**CR**
					CES-D	Felt depressed	0.806	0.042	19.396^**^
						Felt lonely	0.703	0.038	22.601^**^
						Felt sad	0.793	0.051	18.700^**^
						Felt as good as others	0.683	0.052	15.976^**^
						Enjoyed life	0.599	0.051	16.853^**^
						Had poor appetite	0.626	0.051	20.444^**^
						Couldn't get going	0.733	0.052	18.276^**^
						Felt everything was an effort	0.67	0.055	16.746^**^
						Restless sleep	0.623	0.024	11.576^**^
						Felt people were unfriendly	0.450	0.024	13.108^**^
						Felt people disliked me	0.503	0.042	19.396^**^
					CES-D	Depressed	0.953		
						Positive affect	0.926	0.057	15.327^**^
						Somatic	0.615	0.025	10.618^**^
						Interpersonal relations	0.786	0.057	16.727^**^
Depressed	Felt depressed	0.828			Depressed	Felt depressed	0.830		
	Felt lonely	0.727	0.041	20.241^**^		Felt lonely	0.726	0.041	20.193^**^
	Felt sad	0.823	0.037	23.691^**^		Felt sad	0.822	0.037	23.663^**^
Positive affect	Felt as good as others	0.864			Positive affect	Felt as good as others	0.852		
	Enjoyed life	0.724	0.050	16.818^**^		Enjoyed life	0.734	0.051	16.918^**^
Somatic	Had poor appetite	0.673			Somatic	Had poor appetite	0.671		
	Couldn't get going	0.785	0.070	17.226^**^		Couldn't get going	0.786	0.071	17.126^**^
	Felt everything was an effort	0.690	0.068	15.512^**^		Felt everything was an effort	0.695	0.069	15.518^**^
	Restless sleep	0.653	0.070	14.787^**^		Restless sleep	0.648	0.071	14.621^**^
Interpersonal relations	Felt people were unfriendly	0.692			Interpersonal relations	Felt people were unfriendly	0.704		
	Felt people disliked me	0.817	0.101	12.008^**^		Felt people disliked me	0.803	0.099	11.82^**^

B, Non-standardized coefficient; SE, standard error; CR, critical ratio;

** *p < 0.001*.

### Reliability and Correlation of the CES-D-11

As shown in Table 4, overall reliability of the scale was good. Cronbach's α coefficient, according to the four factors, was also good. Reliability coefficients were 0.834, 0.770, 0.791, and 0.722 for depressed, positive affect, somatic, and interpersonal relations, respectively. The correlation coefficients for four factors ranged from 0.371 (between positive affect and interpersonal relations) to 0.722 (between depressed and somatic) and all coefficients were statistically significant (*p* < 0.01).

**Table 4:** Internal Consistency and correlation of the Four-Factor Model of the CES-D-11.

**Category**	**Cronbach's α (95% CI)**	**Depressed**	**Positive affect**	**Somatic**
Depressed	0.834 (0.811 ~ 0.854)	-		
Positive affect	0.770 (0.732 ~ 0.802)	0.591^**^	-	
Somatic	0.791 (0.764 ~ 0.815)	0.722^**^	0.585^**^	-
Interpersonal Relations	0.722 (0.677 ~ 0.761)	0.488^**^	0.371^**^	0.387^**^

CI, confidence interval;

** *p < 0.001*.

## Discussion

The purpose of this study was to investigate the factor structure of the CES-D-11 and verify its validity for PWPD. Due to the potential severity of depression, the ability to detect and treat the condition early, particularly in populations which are at the most risk, is important to researchers and clinicians.

The original psychometric testing of the CES-D suggested a four-factor model (6). In a study on the factor structure of the CES-D-11, Kohout and Berkman (20) reported that it had the same factor structure as the original CES-D-20 (6). Gellis's study (31) surveyed elderly people receiving home health care and found that the CES-D-11 was similarly identified as in Radloff's study (6). However, in a study by Carpenter and Andrykowski (32) comparing the factor structure of the CES-D-11 in six different groups of women, only one group looked at the same factor structure as Radloff (6). There have been some reports of a one-factor structure among English- and Spanish-speaking participants (33) and patients with mental health disorders (34). The results of the present study showed a four-factor structure of the CES-D-11 for PWPD, which is consistent with the original study.

Additionally, the findings indicated that the bifactor CES-D-11 could be used for PWPD. The unidimensionality of the CES-D-11 was confirmed through CFA results for the bifactor model. Utilizing total scores measured using some scales is only possible if the unidimensionality of the scale meets the assumption (35). A CES-D-11 total score of 16 is used as a cutoff for potential depressive symptoms (23). Validated depression measures are required in rehabilitation programs in order to adequately reflect actual differences made by interventions for depression (36, 37) As an instrument's psychometric properties may vary depending on the population, if unidimensionality is not confirmed, a CES-D-11 cutoff score of 16 cannot be used for clinical screening. The results of the present study suggested that a four-factor model is suitable, and that the total CES-D-11 score can be used for PWPD.

Self-report measures should have internal consistency reliability over 0.70 (38); specifically, 0.70 is an acceptable level, 0.80 is good reliability, and above 0.90 is a maximum value for reliability. The results of this study suggested that the CES-D-11 has an adequate level of internal consistency in measuring depression in PWPD. Good reliability for the CES-D has been previously reported, such as an internal consistency of 0.95 in the Greek translation (10). Overall good reliability of 0.88 was reported in patients with systemic sclerosis, and reliability for the specific four factors of depressive affect, somatic/vegetative, interpersonal symptoms, and positive affect were 0.88, 0.80, 0.67, and 0.82, respectively (39). The results of the present study showed acceptable levels of reliability (above 0.70) for each of the four factors.

When using a measurement tool, verification of reliability and validity to determine whether it is measuring the intended variables, without errors, is a prerequisite. Proven measures of depression for PWPD are needed for screening and examining intervention effects. The choice of a measurement method best suited for an application depends on a number of factors: the nature of the study sample, burden on respondents, and management practices, as well as the scale's original intent and psychometric properties. Since psychometric characteristics of a measurement tool may differ according to population, it is necessary to systematically evaluate these characteristics before the widespread use of the tool within a specific population begins (40).

The strength of this study was using panel data with a systematic sampling method. However, there may still be limitations in the samples selected, such as the small size of the younger aged group. The structural elements of the questionnaire may vary according to the characteristics of the sample, such as language or culture. This can make it difficult to draw firm conclusions about whether the four factors are valid for Korean participants with physical disabilities.

In this study, we verified the suitability and reliability of the four-factor CES-D-11 model for PWPD. Considering the high rate of depressive symptoms for PWPD, it is necessary to establish effective support measures for screening, reducing, and preventing depression using a validated tool. The four-factor structured CES-D-11 may be useful in screening for depression in PWPD and verifying the effectiveness of interventions.

## Data Availability Statement

Publicly available datasets were analyzed in this study. This data can be found at: https://www.koweps.re.kr:442/main.do.

## Ethics Statement

Ethical approval was not provided for this study on human participants because Tishe study was based on a secondary analysis of previously collected and publicly available data and therefore an ethics approval was not required as per applicable institutional and national guidelines and regulations. Written informed consent from the participants' legal guardian/next of kin was not required to participate in this study in accordance with the national legislation and the institutional requirements.

## Author Contributions

EY-P has made substantial contribution in interpretation and analysis of data, and drafting the manuscript.

## Conflict of Interest

The author declares that the research was conducted in the absence of any commercial or financial relationships that could be construed as a potential conflict of interest.
